# Role of Na,K-ATPase α1 and α2 Isoforms in the Support of Astrocyte Glutamate Uptake

**DOI:** 10.1371/journal.pone.0098469

**Published:** 2014-06-05

**Authors:** Nina B. Illarionava, Hjalmar Brismar, Anita Aperia, Eli Gunnarson

**Affiliations:** 1 Department of Women’s and Children’s Health, Karolinska Institutet, Stockholm, Sweden; 2 Science for Life Laboratory, Department of Cell Physics, Royal Institute of Technology, Stockholm, Sweden; Universidade de São Paulo, Brazil

## Abstract

Glutamate released during neuronal activity is cleared from the synaptic space via the astrocytic glutamate/Na^+^ co-transporters. This transport is driven by the transmembrane Na^+^ gradient mediated by Na,K-ATPase. Astrocytes express two isoforms of the catalytic Na,K-ATPase α subunits; the ubiquitously expressed α1 subunit and the α2 subunit that has a more specific expression profile. In the brain α2 is predominantly expressed in astrocytes. The isoforms differ with regard to Na^+^ affinity, which is lower for α2. The relative roles of the α1 and α2 isoforms in astrocytes are not well understood. Here we present evidence that the presence of the α2 isoform may contribute to a more efficient restoration of glutamate triggered increases in intracellular sodium concentration [Na^+^]_i_. Studies were performed on primary astrocytes derived from E17 rat striatum expressing Na,K-ATPase α1 and α2 and the glutamate/Na^+^ co-transporter GLAST. Selective inhibition of α2 resulted in a modest increase of [Na^+^]_i_ accompanied by a disproportionately large decrease in uptake of aspartate, an indicator of glutamate uptake. To compare the capacity of α1 and α2 to handle increases in [Na^+^]_i_ triggered by glutamate, primary astrocytes overexpressing either α1 or α2 were used. Exposure to glutamate 200 µM caused a significantly larger increase in [Na^+^]_i_ in α1 than in α2 overexpressing cells, and as a consequence restoration of [Na^+^]_i_, after glutamate exposure was discontinued, took longer time in α1 than in α2 overexpressing cells. Both α1 and α2 interacted with astrocyte glutamate/Na^+^ co-transporters via the 1^st^ intracellular loop.

## Introduction

A tightly regulated intracellular salt homeostasis is of fundamental importance for all mammalian cells, and under physiological conditions most cell types will maintain a fairly stable intracellular sodium concentration ([Na^+^]_i_). This is not true for astrocytes, where fluctuations of [Na^+^]_i_ are regularly occurring. The uptake of glutamate from the synaptic space after neuronal activity, one of the essential functions of the astrocyte, is a major contributor to the astrocytic [Na^+^]_i_ fluctuations [1]. There are five glutamate transporters expressed in the brain [2]. GLAST and GLT-1 are the predominant glutamate transporters in glial cells. Knock-out studies have indicated that glutamate uptake from the extracellular space occurs mainly via the glial glutamate transporters [3], where one glutamate molecule is accompanied by three Na^+^ and one H^+^ in exchange for one K^+^ [4]. In epithelial cells, where fluctuations in [Na^+^]_i_ rarely occur during physiological conditions, the Na^+^-coupled co-transporters, such as the amino acid and glucose co-transporters, generally operate with the stoichiometry ratio 1∶1 or 1∶2 for substrate to Na^+^ [5], [6]. The substrate is delivered in a slow and relatively constant rate, in contrast to the more pulsatile delivery to the astrocyte glutamate co-transporter, following neuronal activity.

Transport via Na^+^-coupled co-transporters is to a large extent driven by the transmembrane Na^+^ gradient. The salt pump, Na,K-ATPase, which actively exports three Na^+^ ions and imports two K^+^ ions for each ATP hydrolyzed, mediates this gradient. Na,K-ATPase exists as a heterotrimeric α/β/γ protein complex, where α is the catalytic ion-transporting subunit [7]. Astrocytes express two α isoforms: α1, which is ubiquitous, and α2, which has more restricted expression. The neurological disorder familial hemiplegic migraine type 2 is caused by mutations in α2 [8]. The functional consequences of the mutations are still incompletely understood.

Studies in cell expression systems have shown that the α2 isoform has a lower Na^+^ affinity than α1 (K_m_ for [Na^+^]_i_ is 12 mM for α1 and 22 mM for α2), and thus α2 will reach V_max_ at a higher [Na^+^]_i_ concentration than α1 [9]. It has been postulated that the high Na^+^ affinity of the ubiquitous α1 isoform will make it less well suited to regulate large influxes of Na^+^. Neurons also express two α isoforms, α1 and α3, and α3 has an almost three-fold lower Na^+^ affinity than α1 [9]. During high neuronal activity [Na^+^]_i_ in postsynaptic structures can increase 20–40 mM [10], and it was recently reported that selective inhibition of α3 almost completely abolishes the capacity to restore [Na^+^]_i_ increases in this range [11]. Pellerin and Magistretti have reported that exposure of cultured astrocytes to glutamate increases Na,K-ATPase activity, and that this effect is to a large extent inhibited by α2-selective ouabain concentrations [12].

Taken together, these findings imply that the Na,K-ATPase α2 isoform is important for the handling and restoring of the transient increases in [Na^+^]_i_ that occur during uptake of glutamate from the synaptic space. To test this concept, we have performed a series of recordings of [Na^+^]_i_ in primary astrocytes, which express both α1 and α2 isoforms and the glutamate/Na^+^ co-transporter GLAST. To examine the specific roles of the α isoforms, studies were performed on astrocytes exposed to isoform-specific ouabain concentrations or on astrocytes overexpressing either α1 or α2. The efficacy of Na,K-ATPase to drive the astrocyte glutamate uptake has also been suggested to be dependent on the interaction between Na,K-ATPase and the glutamate transporters [13], [14].

## Materials and Methods

### Ethics Statement

Animal care and experimental procedures were conducted in accordance with European Communities Council Directive of 24 Nov. 1986 (86/609/EEC). Experimental protocols were approved by the Northern Stockholm Laboratory Animal Review Board (permit numbers N86/09; N426/10; N132/12).

### DNA Constructs Cloning

Coding regions of rat Na,K-ATPase α1 and α2 DNA were amplified using PCR primers (Table S1) and rat brain cDNA as template. PCR fragments were cloned into pENTR/D-TOPO vector (Invitrogen) using GATEWAY TOPO cloning technology (Invitrogen) according to the manufacturer’s protocol. pIRES-mCherry pTurquoise2 and pVenus vectors were made on the base of pIRES-EGFP vector or pEGFP_C vector (Clontech) by substituting EGFP with mCherry or mTurquoise2 (Dr Dorus Gadella provided the plasmid pmTurquoise2-Mito via Addgene plasmid 36208 [15]) or Venus DNA coding sequence using conventional cloning techniques. The Gateway cassette (Invitrogen) was introduced in the SmaI site of the pIRES-mCherry vector and pVenus vector according to the manufacturer’s protocol. The constructs for expression of fluorescent protein mCherry and Na,K-ATPase α1 or α2 subunits from polycistronic mRNA and Venus fusion with Na,K-ATPase α2 were cloned using LR reaction (Invitrogen) with pDEST-IRES-mCherry or pDEST-Venus as destination vectors and corresponding constructs in pENTR/D vector as donors of coding regions for Na,K-ATPase α1 or α2. The superecliptic pHluorin Na,K-ATPase α1 construct DNA was generated by inserting the coding sequence surrounded by flexible linkers (GGGGGS) into the second extracellular loop of Na,K-ATPase (between amino acids 316 and 317). By analogy the Na,K-ATPase α2 was generated by a company (Genscript).

DNA coding regions for Na,K-ATPase α1 and α2 fragments were amplified using PCR primers (Table S1) and rat brain cDNA as template. PCR fragments were conventionally cloned into the EcoRI and XhoI sites of pGEX-6P-1 Glutathione S-transferase (GST) Expression Vector (GE Healthcare) to generate protein expression DNA constructs for pull-down assay.

DNA coding region for GLAST protein fragments was amplified using PCR primers (Table S1) and rat brain cDNA as template. PCR fragment was conventionally cloned into the SacI and SmaI sites of pTurquoise2 vector. The structure of all constructs was confirmed by sequence analyses using universal and coding region specific primers.

### Rat Primary Astrocyte and Neuronal Cell Cultures

Primary cultures of rat astrocytes from striatum and primary cultures of neurons from striatum and hippocampus were prepared from E17 embryonic brains as described previously [16]. Neuronal cultures were prepared in order to obtain neuronal conditioned medium (NCM). Astrocyte cultures were seeded at 2×10^5^ cells/cm^2^ density and grown in DMEM 31885, L-glutamine 1 µM, fetal bovine serum 10%, penicillin-streptomycin 50 µg/ml (all reagents from *GIBCO* Laboratories). Neuronal cultures were seeded at 0.5×10^5^ cells/cm^2^ density and grown in Neurobasal 21203, L-glutamine 1 µM, B-27 supplement, penicillin-streptomycin 50 µg/ml (all reagents from *GIBCO* Laboratories). Astrocytes were cultured for 9–12 days. Neurons were cultured for 15–17 days. For neuronal cultures, half of the culture medium volume was exchanged by fresh medium once a week.

### DNA Transfection

**Figure 1:**
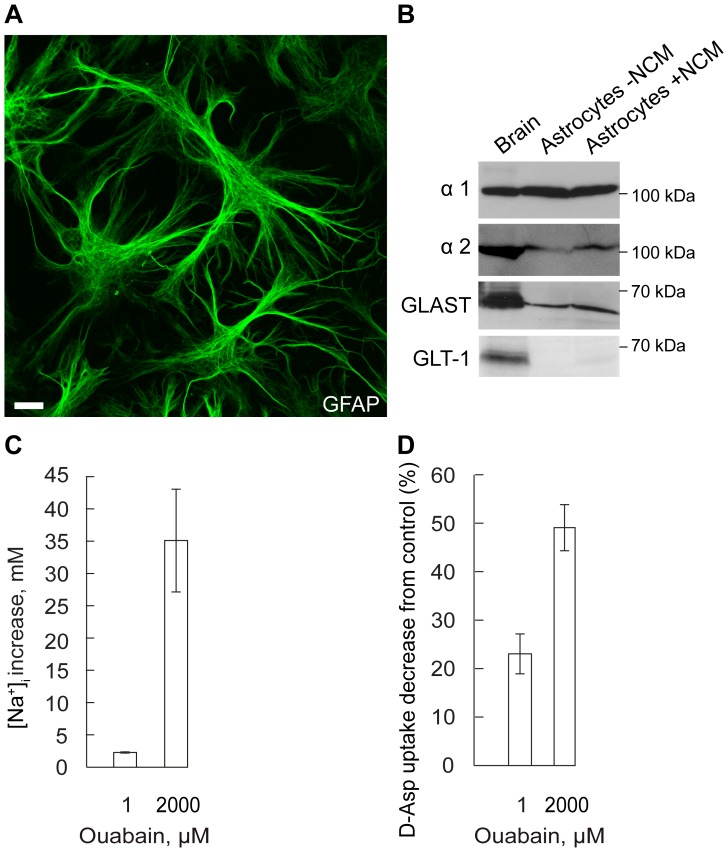
Aspartate uptake, effect of inhibition of Na,K-ATPase α2 and of α2+ α1. **A.** GFAP immunostaining of primary astrocytes with multiple thin processes. Astrocytes were derived from E17 rat striatum and cultured for 9–12 days in medium enriched with neuronal conditioned medium (NCM) during the last 24 h. **B.** Immunoblotting of Na,K-ATPase α1 and α2, GLAST and GLT-1 in whole brain lysate and in primary astrocyte culture treated with NCM (Astrocytes +NCM) or without NCM (Astrocytes -NCM). The expression of the glutamate transporter GLAST in primary astrocyte culture increased by 46% after application of NCM for 24 h (One-way ANOVA, N = 3 experiments, P<0.01). The expression of GLT-1, Na,K-ATPase α1 and α2 did not change. **C.** Increase in astrocyte [Na^+^]_i_ (Δ[Na^+^]_i_) following treatment with ouabain 1 µM or 2000 µM for 15 min (One-way ANOVA, N = 38 cells, P<0.001). **D.** Decrease in D-Asp uptake following treatment with ouabain 1 µM or ouabain 2000 µM for 15 min (One-way ANOVA, N = 9 experiments, P<0.001).

DNA constructs were purified using PureYield Plasmid Midiprep System (Promega) followed by an ethanol precipitation protocol of DNA with Na^+^-acetate [17]. Primary astrocytes were transfected with the respective DNA construct 24 h before [Na^+^]_i_ recordings. Transfection was performed with Lipofectamine 2000 in accordance to manufacturer’s protocol. The transfection reagent was applied for 1 h in Neurobasal medium without antibiotic agents or fetal bovine serum. After transfection, astrocyte cell cultures were returned to the original medium. Mixed hippocampal and striatal (50/50) NCM was added to the astrocyte plate at 1/6 of the total volume. NCM has been shown to increase the expression of glutamate transporters in primary cultured astrocytes [18], [19]. The expression of the glutamate transporter GLAST in our primary astrocyte cultures increased by 46% after application of NCM for 24 h (Fig. 1B and Fig. S1). Actin immunoblot was used for normalization in the calculation of changes in GLAST expression. The expression of GLT-1, Na,K-ATPase α1 and α2 did not change by application of NCM.

### [Na^+^]_i_ Imaging

Measurements were performed using Na^+^-binding benzofuran isophthalate AM indicator (SBFI) (Invitrogen). SBFI was chosen as it is sensitive to [Na^+^]_i_ in the range 0 to 50 mM [Na^+^]_i_ (K_d_ = 11.3 mM). The cover slip with primary astrocytes was washed with Hepes Krebs-Ringer buffer (KCl 4 mM, NaCl 136 mM, Hepes 20 mM, Na_2_HPO_4_*2H_2_0 0.56 mM, KH_2_PO_4_ 0.59 mM, D-glucose 5.6 mM, MgCl_2_*6H_2_O 0.9 mM, NaHCO_3_ 10 mM, CaCl_2_*2H_2_O 1.4 mM) and loaded for 40 min at RT with SBFI 14.7 µM, Pluronic F-127 0.2% (Invitrogen). The cover slip was then mounted into a closed POCmini-2 chamber (PeCon GmbH). Perfusion flow during measurements was estimated to be ∼35 µl/s. Recordings were performed at RT in an upright epifluorescence microscope (Axioscope 2 FS Plus, Carl Zeiss) using a 63×/1.4 NA oil-immersion objective lens, polychrome IV monochromator (Till Photonics) and a cooled CCD camera (ORCA-ERG, Hamamatsu). Fluorescence intensity at 510/30 nm was recorded from alternating 340/15 nm and 380/15 nm excitations with a frame rate 0.2 Hz. Transfected cells were identified by mCherry fluorescence, excited at 543/15 nm and detected with 590 nm long pass filter.

### Cytosolic SBFI Calibration

After SBFI imaging experiments *in situ* SBFI calibration was performed with 0, 5, 10, 15, 20, 40 mM Na^+^ solution. The various Na^+^ solutions were prepared from a 0 mM Na^+^ solution (KCl 27 mM, K-gluconate 136 mM, Hepes 20 mM, KH_2_PO_4_ 0.78 mM, MgSO_4_*7H_2_O 0.8 mM, CaCl_2_ 1.4 mM) and a 50 mM Na^+^ solution (NaCl 27 mM, K-gluconate 114 mM, Na-gluconate 23 mM, Hepes 20 mM, KH_2_PO_4_ 0.78 mM, MgSO_4_*7H_2_O 0.8 mM, CaCl_2_ 1.4 mM). At calibration start, the cells were perfused with a 0 mM Na^+^ solution without ionophores until the fluorescence intensity ratio from the excitations at 340 and 380 nm stabilized. Then a 0 mM Na^+^ solution with ionophores (gramicidin-D 3 µM and monensin 10 µM (Sigma), stock in Dimethyl sulfoxide) and ouabain 1 mM (Sigma) was added, followed by calibration solutions by stepwise increasing Na^+^ concentrations in solutions containing ionophores and ouabain 1 mM. Between the experiments the chamber and perfusion system were decontaminated by perfusion with 95% ethanol twice followed by bi-distilled water. Calculation of [Na^+^]_i_ was performed as described [20]. Briefly, the obtained 340 to 380 normalized intensity ratios (R) for each cell corresponding to the different calibration steps were accurately fit into a three parameter hyperbolic equation model (R^2^>0.95): R = R_0_+((A*[Na^+^]_i_)/([Na^+^]_i_+B)), where R_0_, A and B are coefficients obtained from the fit. Intracellular Na^+^ concentration for each time point was then calculated using equation [Na^+^]_i_ = B*(R–R_0_)/(A+R_0_–R).

### Co-immunoprecipitation

All steps were performed at 4°C, unless stated otherwise. Adult rat brain was homogenized in 10 ml immunoprecipitation buffer (150 mM NaCl, 1 mM EDTA, 100 mM Tris HCl, pH 7.4, 1% Triton X-100, and 1% Na^+^ deoxycholate, protease inhibitors Complete (Roche)). Homogenates were centrifuged 20000 g for 30 min and the pellet was removed. The lysate was pre-cleared by incubation with 300 µl of protein-G agarose beads (Invitrogen) for 1 h with gentle mixing. The supernatant, 4 mg for each sample, was incubated overnight with 8 µg of anti-GLAST, 8 µg of anti-GLT-1 antibody or 8 µg of rabbit IgG for control with gentle mixing. Protein complexes were isolated with protein-G agarose beads by incubation for 2 h with gentle mixing. After centrifugation, the agarose beads were washed six times in immunoprecipitation buffer before elution of bound proteins in 60 µl 2*Laemmli buffer (Tris HCl 0.125 M pH 6.8, β-mercaptoethanol 10%, SDS 4%, glycerol 20%) at 37°C for 30 min, briefly mixed, centrifuged at 1000 g for 2 min and subjected to a 10% sodium dodecyl sulfate polyacrylamide gel electrophoresis (SDS-PAGE).

### GST Pull-down Assay

All steps were performed at 4°C, unless stated otherwise. The *E*. *coli* strain BL21-*DE3* (NEW ENGLAND BioLabs) was transformed with vector pGEX-6p-1 (used for control) or pGEX-6p-1 vector with DNA inserts encoding fragments of Na,K-ATPase α1 or α2. Clones were grown in 50 ml NZYM Broth (Fluka analytical) to the optical density of 0.4 absorbance units measured at 600 nm. Expression of GST fusion proteins were induced with Isopropyl β-D-1-thiogalactopyranoside (Sigma) 0.5 mM and cultures were grown for another 3–4 h at 37°C. Cultures were centrifuged at 4000 g for 15 min, and the bacterial pellet was resuspended in 1.4 ml of radio immuno precipitation assay (RIPA) buffer (Tris-HCl 0.05 M pH 7.4, NaCl 0.150 M, Na-deoxycholate 0.25% (w/v), Triton X-100 1% (v/v), NaH_2_PO_4_ 0.001 M, CaCl_2_ 0.001 M, protease inhibitors Complete (Roche)). The resuspended bacteria were sonicated and centrifuged at 20000 g for 30 min. *E*. *coli* Mach1 (Invitrogen) lysate was prepared from an overnight culture the same way as the *E*. *coli* strain BL21-*DE3* lysate. Glutathione Sepharose 4B (GE Healthcare) 20 µl/sample was washed with RIPA buffer 5 times. To minimize unspecific binding sepharose was blocked for 1 h with *E*. *coli* Mach1 lysate (1 mg/ml) by gentle mixing on a rotator. The total protein concentration was measured for all the *E*. *coli* BL21-*DE3* lysate samples using RC DC Protein Assay and BioRad Dc Protein Assay (BioRad). Eight mg of each BL21-*DE3* lysate was incubated with 20 µl sepharose on a rotator for 2 h at 4°C. The lysates were then centrifuged at 1500 g for 5 min and the supernatant was removed. Forty days old male rat brain was homogenized in 5 ml of RIPA buffer on ice and centrifuged at 20000 g for 30 min. Rat brain lysate was pre-cleared with washed sepharose for 1 h, centrifuged at 20000 g for 5 min and the pellet was removed. The total protein concentration was measured for each rat brain lysate. Each GST fusion protein sepharose sample was incubated with 4 mg of rat brain lysate in 1 ml RIPA buffer overnight. All samples were washed with 1 ml RIPA buffer on a rotator for 5 min and centrifuged at 1500 g for 1 min. A complete supernatant removal was performed with an elongated tip (Prot/Elec Tips-Bulk; BIO RAD). The washing procedure was repeated 6 times. After the last supernatant removal samples were heated in 2*Laemmli buffer (Tris HCl 0.125 M pH 6.8, β-mercaptoethanol 10%, SDS 4%, glycerol 20%) at 37°C for 30 min, briefly mixed, centrifuged at 1000 g for 2 min and subjected to a 10% SDS-PAGE.

### Immunocytochemistry

**Figure 2:**
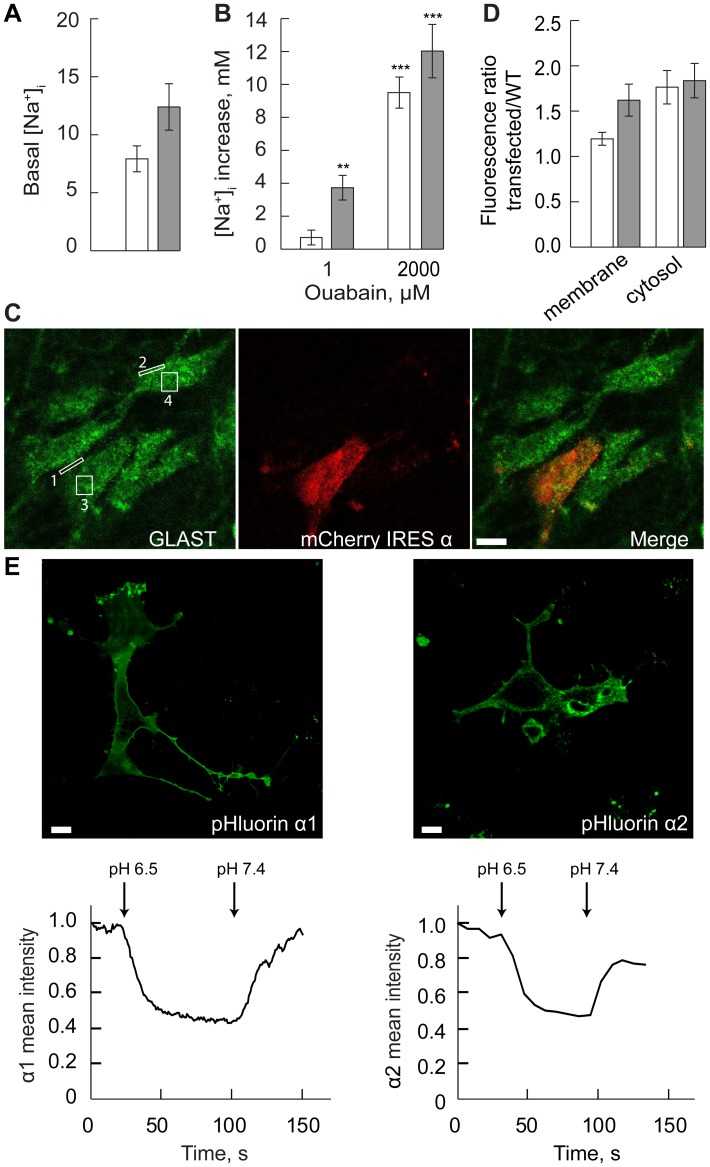
Astrocytes overexpressing Na,K-ATPase α1 or α2, characterization of a model. **A.** Basal [Na^+^]_i_ in astrocytes overexpressing Na,K-ATPase α1 (white) or Na,K-ATPase α2 (grey). **B.** Increase in [Na^+^]_i_ (Δ[Na^+^]_i_) in rat primary astrocytes expressing α1 (white) or α2 (grey) in response to treatment with ouabain 1 µM and 2000 µM for 5 min, respectively (Repeated Measures ANOVA, N = 30 cells, **P<0.01 and ***P<0.001). **C.** Images of immunostaining with GLAST (left image) in primary cultured astrocytes transfected with mCherry IRES Na,K-ATPase α1 or α2 (middle image). Merged image of GLAST and mCherry IRES (right image). Regions of interest were selected in plasma membrane (#1 and #2) and cytosol (#3 and #4) of transfected and non-transfected cells in the same field of view. Ratio of GLAST fluorescence intensities was estimated between region #1/#2 and #3/#4 (for details see Materials and Methods). Values were compared between α1 and α2 transfected cells. Scale bar 10 µm. **D.** GLAST immunofluorescence intensity ratios (transfected vs. non-transfected cells (WT)) in plasma membrane and cytosol of primary astrocytes expressing Na,K-ATPase α1 (white) or α2 (grey). There were no significant differences in GLAST expression between α1 and α2 expressing cells. All data are presented as mean values (bar) and SEM (whiskers). **E.** Primary astrocytes expressing Na,K-ATPase α1 or α2 with an extracellular pH sensitive tag - superecliptic pHluorin. The mean fluorescent signal was measured for the whole cell. The pH dependent fluorescent signal was rapidly attenuated in response to a change of the extracellular pH from 7.4 to 6.5 (curves below images). Reversal of the pH restored the fluorescent signal, indicating that transfected Na,K-ATPase α1 and α2 were inserted in the plasma membrane. Scale bar 10 µm.

Cover slips with cells were washed twice with warm PBS (Na_2_HPO_4_ 7.7 mM, NaH_2_PO_4_ 2.3 mM, NaCl 150 mM). Cells were fixed with 4% paraformaldehyde for 10 min at RT and washed twice with PBS followed by permeabilization with Triton X-100 0.3% 5 min at RT. Fixed cells were incubated with 7% bovine serum albumin (BSA) (Sigma) in PBS for 1 h at RT. Antibodies against GLAST (1∶400, ABCAM) or GFAP (1∶400, Santa Cruz Biotechnology) were used in 3.5% BSA PBS for 2 h at RT. Cover slips were washed three times with PBS and Alexa 488 conjugated secondary antibody (1∶2000, Invitrogen) was applied in 3.5% BSA PBS for 1 h at RT. Cells were washed three times with PBS and mounted. GLAST expression was compared between cells transfected with either α1 or α2. Regions of interest were selected in plasma membrane of transfected cell – region #1 and non-transfected cell – region #2 in the same field of view (Fig. 2C). A ratio of GLAST immunofluorescence intensity was taken between region #1 and region #2 for normalization. The resulting immunofluorescence intensity ratio was compared between α1 and α2 transfected cells. The same comparison was made for cytosol immunostaining in region #3 and region #4 in transfected and non-transfected cells, respectively. In a series of 9 experiments, a total of 18 α1 transfected cells and 16 α2 transfected cells were assessed.

### Cardiotonic Steroids

The α subunit is a specific receptor for the family of cardiotonic steroids. The cardiotonic steroids are to be considered as hormones, i.e. highly specific ligands, which in low (pM-nM) concentrations trigger a signaling capacity of Na,K-ATPase, but which will in higher concentrations inhibit the pumping function of Na,K-ATPase [21], [22], [23]. Ouabain, which has been identified as one of the mammalian cardiotonic steroids, is frequently used as a tool to study Na,K-ATPase activity. High concentrations of cardiotonic steroids inhibit Na,K-ATPase activity. In rodents the response is isoform specific. The IC50 value for ouabain is 48 µM for rat α1 and 58 nM for rat α2 [24]. Ouabain 1 µM will cause an almost full inhibition of rat α2, without any effect on rat α1, and can therefore be used for selective inhibition of α2.

### ^3^H D-Aspartate Uptake

Astrocytes cultured for 9–12 days in a 12 well plate with 18 mm cover slips were washed 3 times with 1 ml Hepes Krebs-Ringer buffer. Hepes Krebs-Ringer 0.5 ml was added containing varying concentrations of ouabain; 1 µM, 2000 µM or *threo*-β-Benzyloxyaspartic acid (TBOA) 500 µM, respectively for 15 min pre-incubation. Then 0.5 ml PBS was added containing D-Aspartate (D-Asp) to a final concentration of 200 µM, ^3^H D-Asp 0.15 µCi/well in addition to ouabain; 1 µM, 2000 µM or TBOA 500 µM, respectively, and incubated for 5 min at RT. Cells were rinsed with 1 ml cold Hepes Krebs-Ringer buffer three times and lysed in 1 ml NaOH 0.1 M. Fifty µl of each well lysate were used for protein concentration measurements using RC DC Protein Assay (BioRad). The samples were analyzed by liquid scintillation counting. CPM values were normalized to the corresponding protein concentrations of the samples. In each experiment an average control sample measurement was normalized to 100% and subsequent sample values estimated in relation to this control.

### Co-expression in Plasma Membrane

Primary astrocytes were transfected as described above with DNA constructs for Venus tagged Na,K-ATPase α1 or α2 expression and mTurquoise2 tagged GLAST expression. Cover slips with cells were washed twice with warm PBS (Na_2_HPO_4_ 7.7 mM, NaH_2_PO_4_ 2.3 mM, NaCl 150 mM). Cells were fixed with 4% cold paraformaldehyde for 1 min at RT and washed twice with PBS and mounted. Imaging was done on a Zeiss ELYRA PS.1 in SIM mode using a 63×/1.4 NA objective. mTurqoise2 was excited with 405 nm and detected at 420–480 nm. Venus was excited with 488 nm and detected at 495–575 nm.

### Immunoblotting

Primary antibodies in PBST (Na_2_HPO_4_ 3.2 mM, KH_2_PO_4_ 0.5 mM, KCl 1.3 mM, NaCl 135 mM, Tween 20 0.05%, pH 7.4) with BSA 3% and NaN_3_ 0.02% were incubated overnight (anti-GLT-1 1:3000, Santa Cruz Biotechnology; anti-GLAST 1∶3000, Santa Cruz Biotechnology; anti-Na,K-ATPase α1 1:10000, Upstate; anti-Na,K-ATPase α2 1:3000, Upstate or for co-immunoprecipitation Santa Cruz SC-16049 Biotechnology; anti-actin 1∶3000, BD Transduction Laboratories). Corresponding secondary antibodies (1∶3000) were incubated in PBST with 5% non-fat milk for 2 h at RT.

### Statistical Analysis

All data are presented as mean values ± SEM. Intracellular Na^+^ recordings, D-Asp uptake data and immunolabeling intensity of pull-down samples were compared using One-way ANOVA. The statistical significance of increases in [Na^+^]_i_ in the ouabain sensitivity tests of transfected cells (Fig. 2B) was estimated using Sign test and Repeated Measures ANOVA. A P-value less than 0.05 was accepted as statistically significant.

## Results

### Effect of Selective Inhibition of Na,K-ATPase α1 and α2 Subunits on Aspartate Uptake

We first examined the relative role of Na,K-ATPase α1 and of α2 for the uptake of aspartate (D-Asp), which occurs via the Na^+^-coupled glutamate transporters by the same mechanism as glutamate. The studies were performed on primary astrocytes, derived from E17 rat embryos and cultured in neuron-conditioned medium that was added 24 h before the experiments (see Materials and Methods). After 9–12 days in culture the cells had developed astrocyte-typical morphology, and exhibited several long processes extending from the soma (Fig. 1A). Expression of GLAST and GLT-1 is developmentally regulated and only the GLAST, but not the GLT-1 glutamate transporter, was expressed in the primary astrocyte cultures when examined by immunoblotting (Fig. 1B). Both the α1 and α2 isoforms of the Na,K-ATPase were expressed in the astrocyte culture (Fig. 1B). Ouabain 1 µM was used to selectively inhibit the α2 isoform and the effect of selective inhibition was estimated by recording [Na^+^]_i_. For this purpose the cells were loaded with the Na^+^ sensitive indicator, Na^+^-binding benzofuran isophthalate (SBFI). Mean basal value for [Na^+^]_i_ was 10±1 mM.

Since the major driving force for glutamate uptake is the transmembrane Na^+^ gradient, we measured both [Na^+^]_i_ and D-Asp uptake in primary astrocytes. Selective inhibition of the α2 isoform produced a small, 2±0.1 mM, increase in [Na^+^]_i_, P<0.001 (Fig. 1C), and a large, 23±4%, decrease in D-Asp uptake. Both effects were significant and reached a P value <0.001 (Fig. 1D). Ouabain 2 mM, which completely inhibits both α1 and α2, caused a 35±8 mM increase in [Na^+^]_i_ and a 49±5% decrease in D-Asp uptake (Fig. 1C and 1D). The unspecific inhibitor of glutamate transporters, TBOA (500 µM), caused a 76±2% decrease in D-Asp uptake (data not shown).

### Intracellular Sodium in Astrocytes Expressing Predominantly Na,K-ATPase α1 or α2 Isoforms

The relative efficiency of the α1 and α2 subunits to regulate the increases in astrocyte [Na^+^]_i_ that accompanies transient increases in extracellular glutamate was studied in primary astrocytes which overexpressed either the α1 or the α2 isoform. To identify transfected cells, the Na,K-ATPase α subunits were co-expressed with the red fluorescent protein mCherry. Mean [Na^+^]_i_ was numerically, but not significantly lower in α1 than in α2 expressing cells (8±1 mM in α1 and 12±2 mM in α2, respectively) (Fig. 2A). Ouabain 1 µM, that selectively inhibits α2, caused a significant increase in [Na^+^]_i_ in α2 expressing cells, but not in α1 expressing cells (Fig. 2B). Ouabain in the non-selective concentration 2 mM significantly increased [Na^+^]_i_ in both α1 and α2 expressing cell. These results supported the assumption that the cells did predominantly express either α1 or α2. To ensure that the transfection procedure would not affect GLAST expression, cells were immuno-stained and GLAST expression was assessed by determining the ratio between the GLAST immunofluorescent signal in transfected and non-transfected cells in corresponding cell domains (Fig. 2C and 2D). No significant difference in expression of GLAST was found between α1 and α2 overexpressing astrocytes (Fig. 2D). To test whether the isoforms were properly inserted in the plasma membrane, we expressed α1 and α2 that were tagged in the extracellular domain with a pH sensitive fluorescent probe in the primary astrocytes. The pH sensitivity was here used to identify proteins inserted in the plasma membrane, since proteins that are not in the plasma membrane but in vesicles will be non-fluorescent due to an acidic environment [16]. In addition, only proteins inserted in the membrane will contribute to the fast response in fluorescent signal seen after changes of extracellular pH. A fluorescent signal that was rapidly attenuated when extracellular pH was lowered to 6.5 and rapidly restored when pH was restored to 7.4 was observed both in cells overexpressing α1 and α2 isoforms. These observations indicate that both α1 and α2 are expressed in the plasma membrane (Fig. 2E).

**Figure 3:**
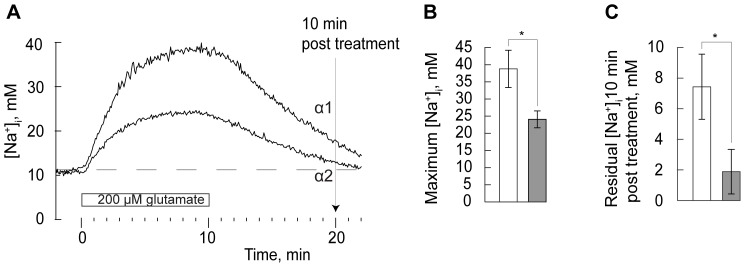
Transient changes in [Na^+^]_i_ following exposure to glutamate 200 µM in astrocytes expressing predominantly Na,K-ATPase α1 or α2 isoforms. A. Astrocyte [Na^+^]_i_ (mean) in Na,K-ATPase α1 and Na,K-ATPase α2 expressing cells exposed to glutamate 200 µM for 10 min (indicated by horizontal white bar). B. Maximum [Na^+^]_i_ after 10 min exposure to glutamate 200 µM in α1 (white) and α2 (grey) expressing astrocytes (One-way ANOVA, N = 18 cells, *P<0.05). C. Residual [Na^+^]_i_ measured at 10 min (indicated with an arrow in Fig. 3A) after discontinuation of glutamate treatment in α1 (white) and α2 (grey) expressing astrocytes (One-way ANOVA, N = 18 cells, *P<0.05).

To examine the effect of glutamate on [Na^+^]_i_ in astrocytes overexpressing either α1 or α2, cells were continuously perfused with a Hepes Krebs-Ringer solution to which 200 µM glutamate was added, a concentration which roughly corresponds to the glutamate levels reached following high neuronal activity [25]. Glutamate was added for 10 min, while [Na^+^]_i_ was continuously recorded in the soma. Exposure to glutamate caused an immediate increase in [Na^+^]_i_ in both α1 and α2 overexpressing astrocytes (Fig. 3A). We attribute the increase in [Na^+^]_i_ to influx of sodium via the glutamate/Na^+^ co-transporter. Notably, the increase was less pronounced in the α2 than in the α1 over-expressing cells. The α1 isoform has a higher sodium affinity than the α2 isoform and is expected to reach at V_max_ at around 25 mM Na^+^, as compared to 40 mM for α2 [9]. The capacity of α1 to counteract the influx of sodium, once the [Na^+^]_i_ exceeds the V_max_, will therefore be limited, and may have contributed to the significantly higher plateau value for [Na^+^]_i_ in the α1 overexpressing cells (∼39 mM) than in α2 overexpressing cells (∼24 mM) (Fig. 3B). As a result, it took longer time for α1 expressing cells to return to the basal [Na^+^]_i_, and 10 min after discontinuation of the glutamate exposure the residual [Na^+^]_i_ was significantly lower in α2 than in α1 overexpressing cells (Fig. 3C).

### Interaction between Na,K-ATPase α1 and α2 and Glutamate Transporters GLT-1 and GLAST

**Figure 4:**
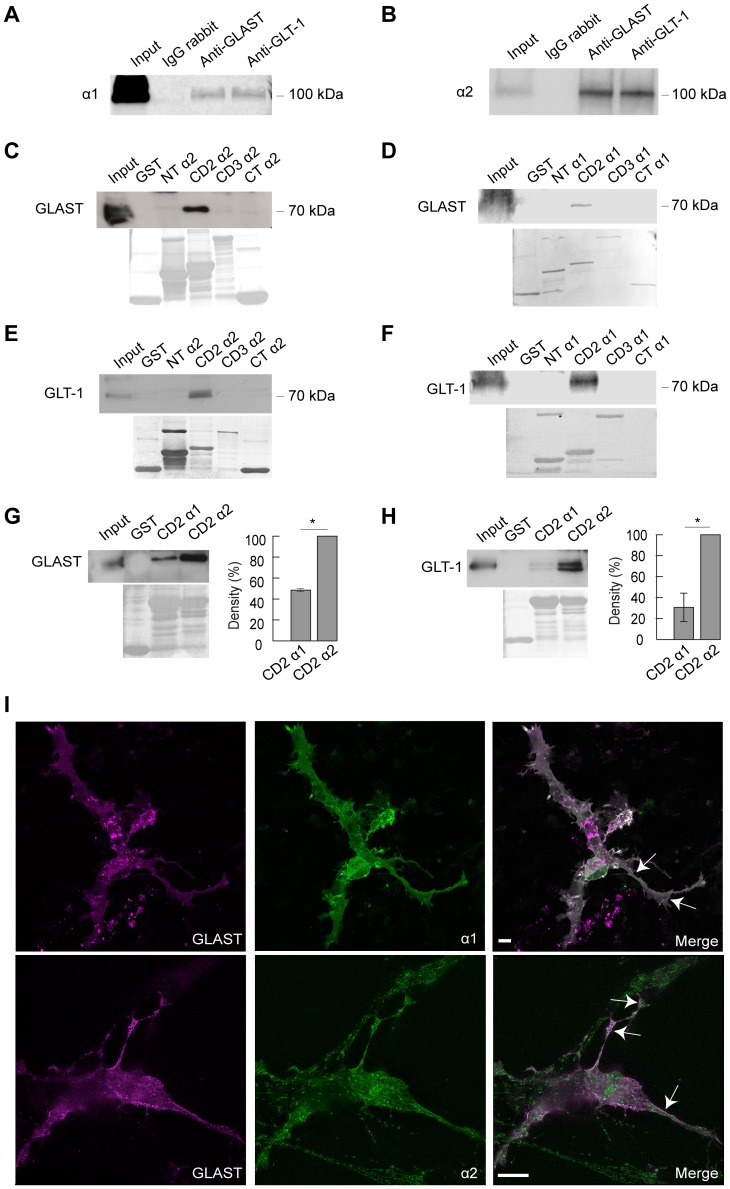
Na,K-ATPase α1 and α2 subunit interaction with astrocyte glutamate transporters GLT-1 and GLAST. **A and B.** GLAST and GLT-1 were co-immunoprecipitated with Na,K-ATPase α1 and α2 from adult rat brain lysate. GLAST and GLT-1 interact with full length Na,K-ATPase α1 (A) and Na,K-ATPase α2 (B). **C–H:** Pull-down assays of GST and GST fused to the Na,K-ATPase α N-terminus (NT), cytosolic domains corresponding to first and second intracellular loops (CD2 and CD3) and C-terminus (CT), respectively, incubated with adult rat brain lysate after immobilization on GST-sepharose. The pull-down probes were subjected to SDS-PAGE and Western blot and probed with antibodies against GLAST or GLT-1. **C and D.** GLAST interacts with the CD2 domain of Na,K-ATPase α2 (C) and Na,K-ATPase α1 (D) but not with NT, CD3 or CT domains of either α1 or α2. **E and F.** GLT-1 interacts with the CD2 domain of Na,K-ATPase α2 (E) and Na,K-ATPase α1 (F) but not with NT, CD3 or CT domains of either α1 or α2. **G and H.** Comparison of the CD2 α1 and α2 interaction between GLT-1 and GLAST. Bar diagram shows mean integrated densities for GLAST (G) and GLT-1 (H) immunostaining in GST pull-down assays of CD2 α1 and CD2 α2, respectively, in percent of CD2 α2 staining density. (One-way ANOVA, N = 3 experiments, *P<0.05). **I.** Co-expression of GLAST and Na,K-ATPase α subunits in primary astrocytes. Primary astrocytes transfected with GLAST (magenta) and Na,K-ATPase α1 or α2 (green). Note co-expression (white) in plasma membrane compartments, including astrocyte processes, indicated by arrows. Scale bar 10 µm.

Three protocols were used to study the preconditions for interaction between the Na,K-ATPase α subunits and the glutamate transporters in astrocytes. Co-immunoprecipitation and pull-down studies were performed using brain tissue from adult rats, while imaging of the localization of α subunits and GLAST were performed on primary astrocytes. The glutamate transporters GLAST and GLT-1 co-precipitated with both α1 and α2 (Fig. 4A and B). To further analyze this interaction, pull-down studies were performed, using peptides corresponding to the α1 or α2 N-terminus (NT), first intracellular loop (CD2), second intracellular loop (CD3) and C-terminus (CT). The intracellular segments of the Na,K-ATPase α1, α2 and α3 isoforms are well conserved while the NTs are more heterogeneous. The CD2 domains from the α1 and from the α2 isoform were found to interact with both glutamate transporters (Fig. 4C, D, E and F). Interaction appeared to be stronger for the α2 than for the α1 isoform (Fig. 4G, H) and the α3 isoform (data not shown). The NT, the CD3 and the C-terminus from α1 and α2 did not interact with either GLT-1 or GLAST.

To estimate the capacity of the α subunits and GLAST to co-localize in primary astrocytes, they were fused to the fluorescent proteins Venus and mTurquoise2, respectively, and expressed in the primary astrocytes. Confocal images show co-expression of α2 and GLAST as well as of α1 and GLAST both in the cell body and in the astrocyte processes (Fig. 4I).

## Discussion

One of the main functions of astrocytes is the uptake of glutamate, released after activation of excitatory synapses. The transmembrane Na^+^ gradient generated by the Na,K-ATPase is a major determinant of glutamate uptake. Data from the current study indicate that both isoforms of the Na,K-ATPase catalytic α subunits are important to support the glutamate transport and that the α2 isoform, which in contrast to the ubiquitous α1 isoform is expressed in only a few cell types, may play an important supportive role for Na^+^ homeostasis in astrocytes due to its relatively low Na^+^ affinity.

It is well recognized that the Na^+^ transients in astrocytes, evoked by the uptake of glutamate, will have an impact on astrocyte metabolism and astrocyte signaling pathways. Yet few studies have been devoted to the role of astrocyte Na,K-ATPase, although it is generally agreed that this enzyme plays a pivotal role for the recovery of glutamate triggered Na^+^ transients. An important reason for this may be that gene knock-out of the ubiquitous α1 results in early embryonic death [26], [27]. Knock-out of the α2 subunit results in perinatal death. Heterozygous α2 knock-out mice are viable [27], [28]. Use of inhibitors is generally not considered to provide conclusive evidence, since most inhibitors are not specific. Fortunately this is not the case for ouabain, which is a steroid hormone, produced in the adrenal gland and the hypothalamus. Ouabain belongs to the family of cardiotonic steroids, consisting of a steroid core, a lactone ring and a sugar moiety. Cardiotonic steroids are highly specific Na,K-ATPase ligands with a dose-dependent inhibition of the pumping activity, and in lower concentrations also trigger a signaling function of Na,K-ATPase. The cardiotonic steroids have as yet not been found to bind to any other mammalian protein. Because the α2 isoform is much more sensitive to the inhibitory effect of ouabain than α1, IC50 values are 58 nM for rat α2 and 48 µM for rat α1 [24], the relative contribution of the two isoforms in maintaining [Na^+^]_i_ can be estimated by performing studies in the presence or absence of 0.1–1.0 µM ouabain. As an additional approach to differentiate between the function of α1 and α2, we used cells that overexpressed either α1 or α2 and measured the response to glutamate triggered Na^+^ transients. The astrocytes were transfected with either α1 or α2, but not with the Na,K-ATPase β subunit.

The two catalytic isoforms of Na,K-ATPase, α1 and α2, were both endogenously expressed in primary astrocytes. This allowed us to study the relative importance of each endogenous isoform as a driving force for glutamate uptake. Selective inhibition of endogenous α2 caused a relatively large (23%) inhibition of glutamate transport in relation to a very modest (2 mM) increase in global [Na^+^]_i_. As the global [Na^+^]_i_ was recorded mainly from the cell soma, this finding raises the question whether global [Na^+^]_i_ gradients might exist and whether astrocyte processes may provide restricted space for diffusion of Na^+^. Glutamate transporters are not evenly expressed in the astrocyte plasma membrane, but are concentrated in the processes facing nerve terminals, axons and spines [29].

Two recent studies have provided evidence for an interaction between the Na,K-ATPase α catalytic subunits and the glutamate transporters in astrocytes [13], [14]. In the study by Rose et al. [14], interaction of the α2 subunit with GLAST and GLT-1 was found using co-purification and co-immunoprecipitation methods. Results from immunostaining studies were also compatible with co-localization. This group found little expression of the ubiquitous α1 in astrocytes. In a study by Genda et al. [13] chromatography-coupled tandem mass spectrometry was used to identify proteins interacting with the GLT-1 transporter. The Na,K-ATPase α1, α3 and the β1 subunit, but not the α2 subunit, were identified. Interaction with α1 and α3 were also found in co-immunoprecipitation studies. Since co-immunoprecipitation studies are dependent on highly selective antibodies, we also examined the interaction between the glutamate transporters and GST-fused segments of α1 and α2 subunits. Both glutamate transporters GLAST and GLT-1 were found to interact with the segment corresponding to the first intracellular loop CD2 of the α1 and α2 subunits, but the interaction with CD2 of the α2 subunit appeared to be stronger. The CD2 constitutes the major proportion of the actuator (A) domain of the α subunit [30] and has a high homology between isoforms and between species. GLAST and GLT-1 have been reported to overlap with α2 in astrocyte processes surrounding mostly glutamatergic synapses [31]. We observed a stronger interaction between the first intracellular loop of α2 and GLAST than between the first intracellular loop of α1 and GLAST. This, together with the finding that in primary astrocytes, that only expressed the endogenous α isoforms, specific inhibition of α2 resulted in a relatively small increase in [Na^+^]_i_ and a relatively large decrease in aspartate uptake (used as an indicator for glutamate uptake), may imply a more specific co-localization and functional interaction of α2 and GLAST than between α1 and GLAST.

Abnormally high glutamate concentration in the synaptic space is a major cause of neuronal injury via excitotoxicity. Efficient uptake of glutamate into astrocytes via the glutamate/Na^+^ co-transporters depends on the transmembrane Na^+^ gradient generated by Na,K-ATPase [32]. The ambient glutamate concentration is around 25 nM, but can increase to levels in the range of 200 µM–1 mM following neuronal activity [25], [33]. The released glutamate needs to be rapidly cleared from the extracellular space to avoid detrimental effects of inappropriate activation of glutamate receptors. This clearance of glutamate is critically dependent on the capacity of the astrocyte Na^+^-coupled glutamate transporters and is, due to the stoichiometry of one glutamate to three Na^+^ ions, accompanied by transient increases in astrocyte [Na^+^]_i_ [34], [35]. To accommodate repeated release of glutamate [Na^+^]_i_ needs to be rapidly restored. The results from the present studies on α1 and α2 overexpressing astrocytes suggest that α2 may be more efficient than α1 to rapidly restore a large transient increase in [Na^+^]_i_. Exposure to glutamate 200 µM caused a larger increase in [Na^+^]_i_ in α1 than in α2 overexpressing cells and, as a consequence, the recovery time for [Na^+^]_i_ was shorter in cells overexpressing α2. These findings may imply that the Na^+^ transients that accompany the uptake of glutamate released by neuronal activity could exceed the V_max_ for α1, while α2 will continue to pump efficiently during the entire exposure time. As a consequence, the recovery time can be prolonged if the astrocyte would only express α1.

The absolute number of α1 and α2 isoforms expressed in the transfected cells could not be determined, due to methodological limitations. An alternative explanation for the higher efficacy of α2 over-expressing cells to handle the glutamate-associated transient increase in [Na^+^]_i_ might therefore be that more pumps are expressed in the plasma membrane in the α2 than in the α1 over-expressing cells. Indirect evidence, including the fact that the endogenous α1 isoform had not been down-regulated, makes this interpretation less likely.

It should be emphasized that much of the importance of this study lies in that it raises the question whether astrocytes need to express two NKA α isoforms not only because α1 and α2 may differ with regard to subcellular localization and capacity to interact with the glutamate transporters, but also because of their differences in sodium affinity. Emerging evidence suggests that [Na^+^]_i_ has a signaling function in astrocytes, which affects the metabolic processes that control production of lactate [36], [37], [38]. Even small changes in the Na^+^ gradient across the plasma membrane have consequences for astrocyte metabolism and signaling, since they may reduce the driving force for other Na^+^-coupled transport functions such as glucose uptake. The functional differences between the Na,K-ATPase α1 and α2 isoform described in this study may also have implications for the understanding of astrocyte associated pathology and dysfunction in disease.

## Supporting Information

Figure S1 **GLAST expression in primary astrocyte culture.** Immunoblotting of GLAST and actin in whole brain lysate and in primary astrocyte culture treated with NCM (+NCM) or without NCM (−NCM). The expression of the glutamate transporter GLAST in primary astrocyte culture increased by 46% after application of NCM for 24 h, mean integrated density is shown in a bar diagram (One-way ANOVA, N = 3 experiments, P<0.01).
(TIF)

Table S1 (TIF)
