# Correction: Role of Na,K-ATPase α1 and α2 Isoforms in the Support of Astrocyte Glutamate Uptake

**DOI:** 10.1371/journal.pone.0110095

**Published:** 2014-09-29

**Authors:** The PLOS ONE Staff

The first author’s name is spelled incorrectly. The correct name is: Nina B. Illarionova. The correct citation is: Illarionova NB, Brismar H, Aperia A, Gunnarson E (2014) Role of Na,K-ATPase α1 and α2 Isoforms in the Support of Astrocyte Glutamate Uptake. PLoS ONE 9(6): e98469. doi:10.1371/journal.pone.0098469
