# Supplementary figures and images for: Role of Na,K-ATPase α1 and α2 Isoforms in the Support of Astrocyte Glutamate Uptake
Source: PLoS One. 2014 Jun 5;9(6):e98469. doi: 10.1371/journal.pone.0098469 (PMC4046997; doi:10.1371/journal.pone.0098469)

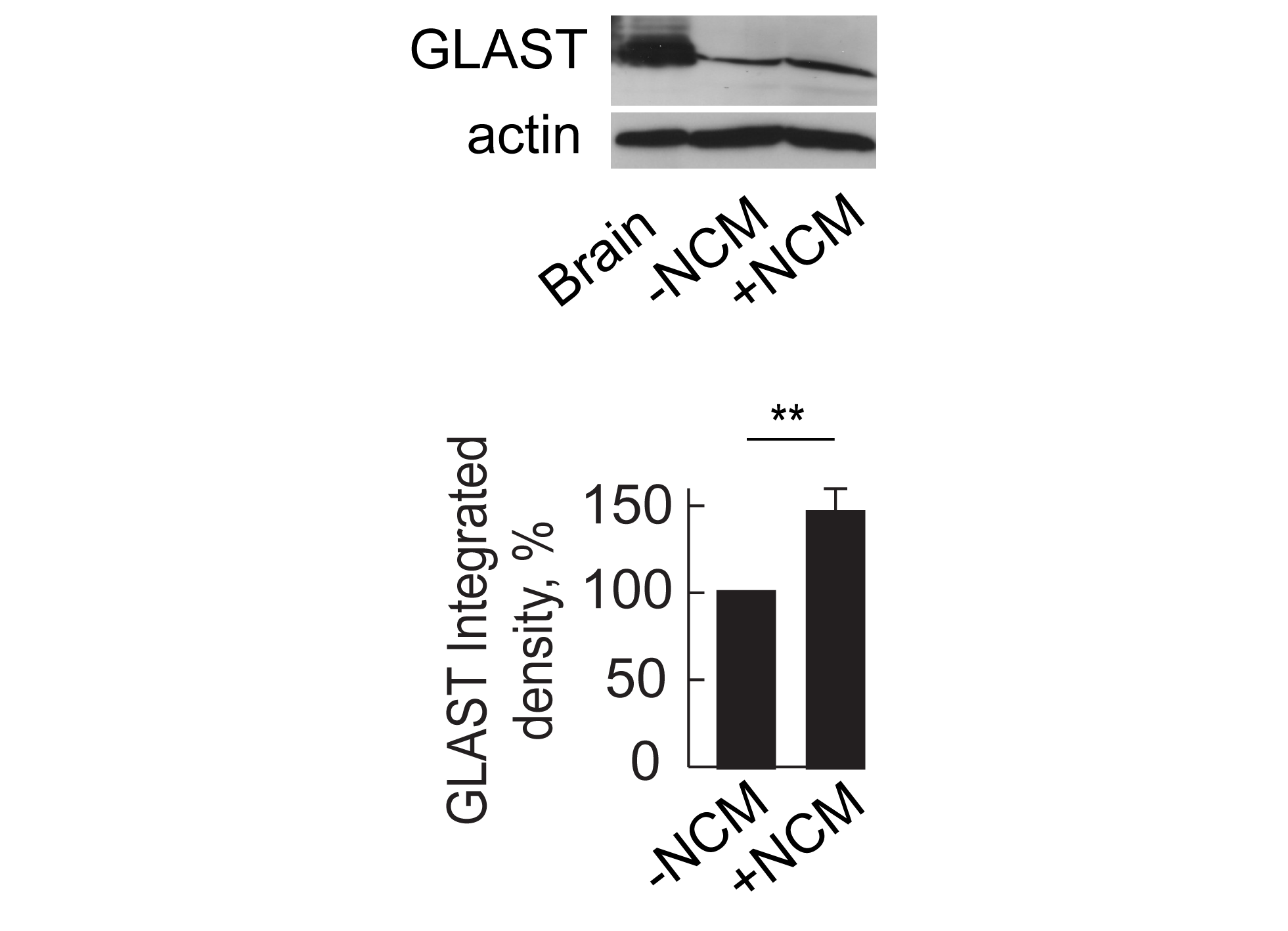

Supplement: Figure S1 — GLAST expression in primary astrocyte culture. Immunoblotting of GLAST and actin in whole brain lysate and in primary astrocyte culture treated with NCM (+NCM) or without NCM (−NCM). The expression of the glutamate transporter GLAST in primary astrocyte culture increased by 46% after application of NCM for 24 h, mean integrated density is shown in a bar diagram (One-way ANOVA, N = 3 experiments, P<0.01). (TIF) [file pone.0098469.s001.tif]

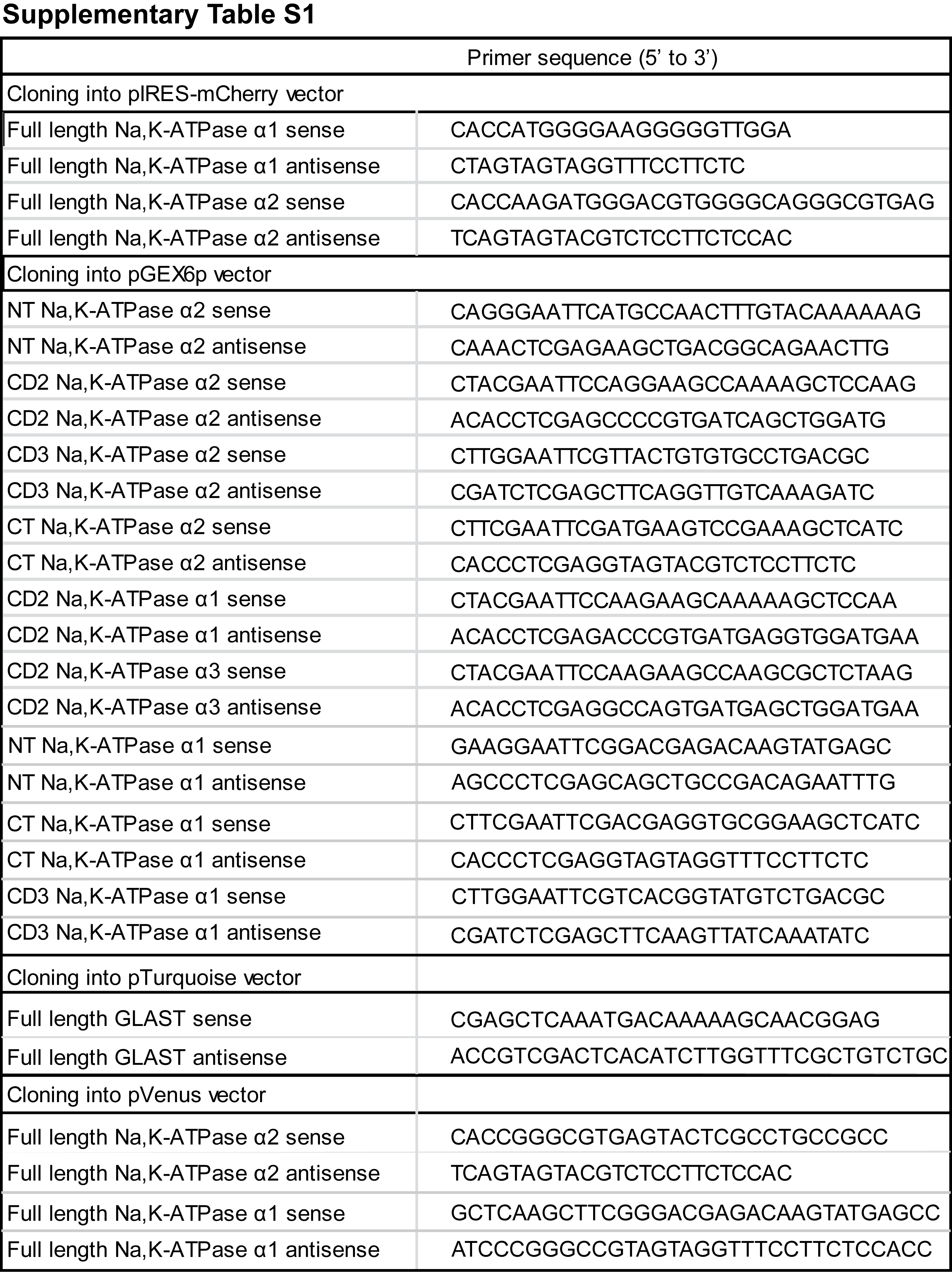

Supplement: Table S1 — (TIF) [file pone.0098469.s002.tif]
